# Large B-cell lymphoma in a dog: A cyto-histopathological evaluation and Immunophenotyping according to WHO classification for canine lymphomas

**Published:** 2016-03-15

**Authors:** Zahra Nikousefat, Mohammad Hashemnia, Moosa Javdani

**Affiliations:** 1 *Department of Clinical Sciences, School of Veterinary Medicine, Razi University, Kermanshah, Iran;*; 2 *Department of Pathobiology, School of Veterinary Medicine, Razi University, Kermanshah, Iran;*; 3 *Department of Clinical Sciences, Faculty of Veterinary Medicine, Shahrekord University, Shahrekord, Iran.*

**Keywords:** Canine lymphomas, Cyto-histopathological features, Immunohistochemistry

## Abstract

In the present study, we described cyto-histopathological features and immunophenotyping of the large B-cell lymphoma in an 8-year-old mixed breed dog with applying the World Health Organization (WHO) system of classification of canine lymphomas. In fine-needle aspiration (FNA), lymph nodes were involved by neoplastic cells of intermediate to large size with deep blue cytoplasm; consist of centroblasts, immunoblast and medium-sized cells. Histopathologically, the follicles and sinuses of lymph nodes were replaced by sheets of numerous immunoblasts (less than 90.0% of total cells) and centroblasts. Numerous mitotic figures were also observed. Immunohistochemical analysis presented that the neoplastic cells express B-cell phenotype CD20 and CD79a, but do not stain for T phenotype CD3. On the basis of cytology, histopathology and immunohistochemical findings, the present tumor was diagnosed as diffuse large B-cell lymphoma, high-grade centroblastic type (DLBCL-CB) according to WHO histological classification. Applying this classification system for diagnosis of canine lymphomas is very useful and has a high accuracy and consistency. However, further co-operative studies between clinicians and pathologists should be performed, in order to improve the effectiveness of this classification.

## Introduction

Lymphoma, also referred to as lymphosarcoma or malignant lymphoma, is the most common hematopoietic neoplasm in the dogs, with an incidence approaching 0.1%; 80.0% of lymphomas are seen in 5-11-year-old dogs. Lymphoma arises due to the proliferation of malignant lymphoid cells usually in lymphoid tissues, such as lymph nodes, spleen, thymus or mucosa-associated lymphoid tissue (MALT), but the tumor may originate in practically any tissue. This origin from solid organs distinguishes lymphoma from lymphoid leukemia, as the latter arises from bone marrow.^1^^,^^2^

The precise etiology of lymphoma in the dog has not been identified, although several hypotheses have been investigated but none definitely proven. Suggested etiologies for canine lymphoma include retroviral infection, environmental contamination with herbicides (2,4-dichlorophenoxyacetic acid), magnetic field exposure, chromosomal abnormalities, and immune dysfunction. There are four different anatomical forms: multicentric (characterized by generalized lymphadenopathy), alimentary (characterized by infiltration of the gastrointestinal tract and/or gastrointestinal lymph-adenopathy), mediastinal (characterized by generalized lymphadenopathy) and extranodal (affecting any other organ or tissue).^1^^,^^2^

Despite great progress in identifying the various aspects of canine lymphoma, the classification, treatment and prognosis of these tumors are major challenges for veterinarians. Many attempts have been made to identify tumor and patient factors that may act as useful predictors of response and prognosis for canine lymphoma. Patient factors such as age, sex, body weight and breed have all at some time been suggested to be of importance but none has consistently correlated with prognosis. The clinical stage of the tumor does appear to have some bearing on the outcome. Tumor-related factors such as mitotic index (MI), AgNOR counts and immunophenotype have also been the subject of many studies. The introduction of multi-agent chemotherapy protocols for canine lymphoma made this disease treatable, if not curable.^3^^,^^4^

Currently, lymphomas in dogs are treated regardless of their types, but researchers now find that the canine lymphomas are of many types and the treatment of lymphoma can be more effective when specific identification is done. The World Health Organization (WHO) has devised a new system of recognizing and categorizing the many subtypes of human lymphoid tumors with very different characteristics that must be considered in providing effective treatments.^5^^,^^6^

In the present paper, we described a multicentric lymphoma in a dog and also classified lymphoma into a distinct entity according to a modified WHO classification of canine lymphomas recently published by Valli *et al*.^7^

## Materials and Methods

**Case description. **An 8-year-old male mixed breed dog was referred for evaluation of loss of appetite. On clinical examination, the dog was alert with generalized peripheral lymphadenomegaly and diffuse splenomegaly on abdominal palpation. 

**Cytological evaluation. **Cytological smears obtained by Fine-needle aspiration (FNA) of a lymph node were air-dried, fixed, and stained with the May-Grunwald-Giemsa (MGG) procedure. The morphological classification criteria according to Fournel-Fleury *et al*.^8^ and Ponce *et al*. ^9^ were based on cell size, ‘small’, ‘medium’ or ‘large’, i.e. the nucleus smaller than, equal to, or larger than two red blood cells (RBCs), respectively, the shape of the nucleus, the density of the chromatin, the number, size and distribution of the nucleoli, and the extension and basophilia of the cytoplasm.

**Histopathological evaluation. **Biopsy specimens of enlarged lymph nodes were fixed in 10.0% neutral buffered formalin and embedded in paraffin wax. Sections were cut and stained with hematoxylin and eosin (H & E) for micro-scopic examination. Nuclear size was determined as small (< 1.5 fold the size of a RBC), intermediate (1.5 to 2 fold the size of a RBC), or large (> 2 fold the size of a RBC). The mitotic rate was quantified in histological specimens by scanning 10 fields at 40× lens and counting the mitotic figures. The mitotic rate was counted in areas having the highest number of mitotic figures. Lymphomas with 0 to 5 mitoses per 400× field were graded as low grade, with 6 to 10 mitoses per 400× field as medium grade, and those with greater than 10 mitoses per 400× field as high grade. The diagnosis was made according to WHO histological classification.

**Immunological evaluation. **Our case was labeled immunohistochemically for B and T cell antigens with human antibodies cross-reacting with canine leukocyte on paraffin sections. Immunostaining was performed using CD3, CD20 and CD79a (DAKO, Copenhagen, Denmark). Briefly, tissue sections were cooked at 60 ˚C and then deparaffinized in xylene. In order to antigen retrieval, the slides were inserted in Tris-EDTA, heated and then cooled. The slides were put in Tris buffer and hydrogen peroxide 3.0% plus methanol was used for endogenous peroxidase activity inhibition. The sections were incubated with selected monoclonal antibodies. Envision solution was used and then diaminobenzidine chromogen was used as a substrate. The sections were stained with hematoxylin. In the next step, they were washed with distilled water and then dehydrated with grading alcohol. Then, the Tris buffer solution was used for washing.

**Animal ethics. **This study was performed under the approval of the state committee on animal ethics, Razi University, Kermanshah, Iran. Also, the recommendations of European Council Directive (86/609/EC) of November 24, 1986, regarding the protection of animals used for experimental purposes, were considered.

## Results

**Fig. 1:**
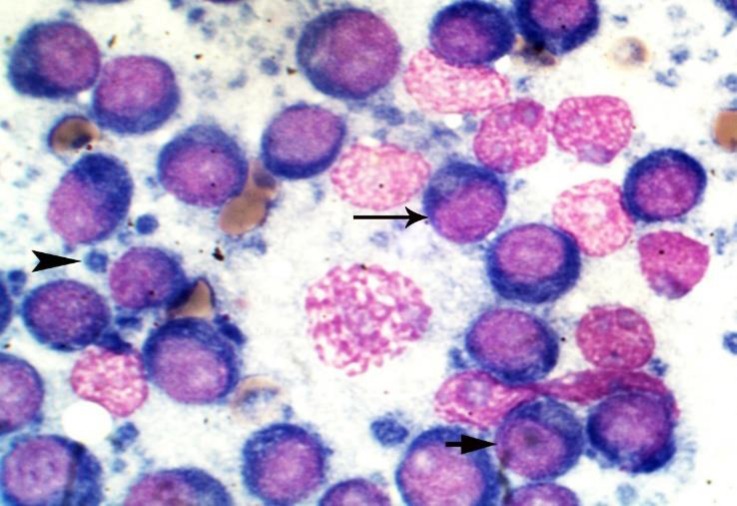
Fine-needle aspiration of lymph nodes; neoplastic cells consist of centroblasts (arrow), immunoblast (short arrow) and intermediate cells; lymphoglandular bodies are seen (arrowhead), (MGG staining, 2500×).

**Cytological findings. **In FNA, reactive lymph nodes were involved by neoplastic cells of intermediate to large size with deep blue cytoplasmand variable nuclear size and shape consist of centroblasts, immunoblast and medium-sized cells. Few mitotic figures were identified (Fig. 1). The neoplasm was diagnosed as centroblastic polymorphic lymphoma: predominantly large cell according to the updated Kiel classification of the canine lymphomas and large B cell lymphoma, centroblastic type according to the WHO classification.^10^^,^^11^

**Fig. 2:**
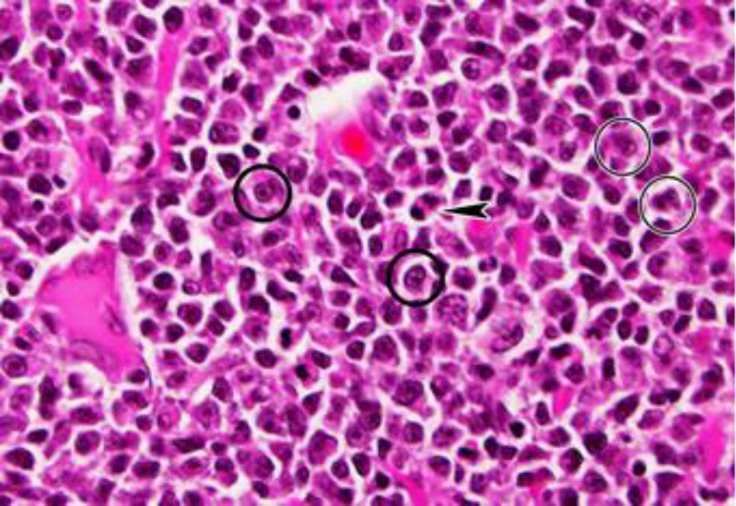
Micrograph of the lymph node; follicles and sinuses are replaced by sheets of numerous immunoblasts and centroblasts. Immunoblasts contain small to large nuclei and centrally prominent nucleoli (thick circles); centroblasts, have a moderate amount of cytoplasm and large, round nuclei with multiple nucleoli (thin circles). Mitotic figures are seen (arrowhead), (H & E; 1000×).

**Histopathological findings. **The normal architectural features of the involved lymph node, including the follicles and sinuses, were replaced by sheets of numerous immuno-blasts (less than 90.0% of total cells) and centroblasts (Fig. 2). 

There were also many cells with morphological features intermediate between centroblasts and immunoblast with blastoid chromatin. The size of immunoblasts nuclei was varying from small to large. The most lymphoma cells, which resemble centroblasts, had a moderate amount of cytoplasm and large, round nuclei with multiple nucleoli. In this case, MI was 13 which estimated as high grade. The histopathological diagnosis was diffuse large B cell lymphoma, high-grade centroblastic type (DLBCL-CB) according to WHO histological classification. 

**Fig. 3:**
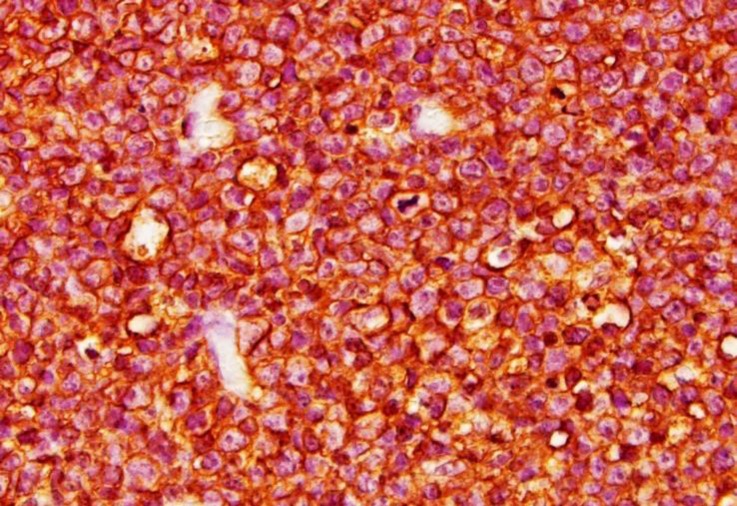
Micrograph of the lymph node; neoplastic cells are positive by immunolabeling with B-cell phenotype CD20, (IHC staining; 1000×).

**Fig. 4:**
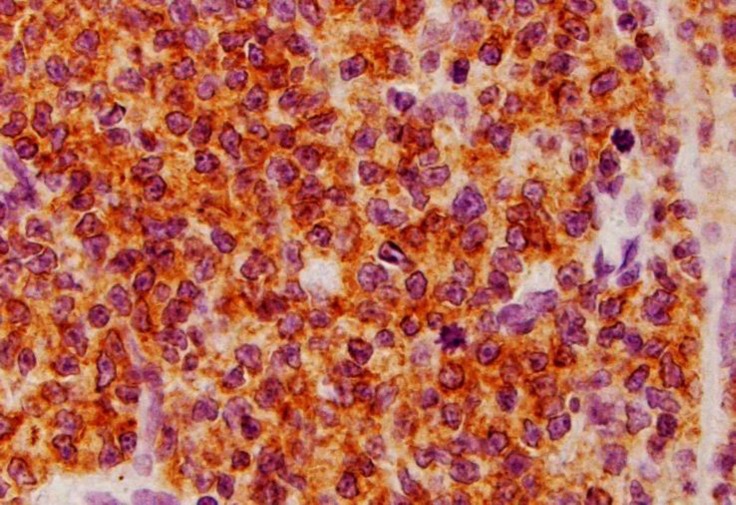
Micrograph of the lymph node; neoplastic cells are positive by immunolabeling with CD79a, (IHC staining; 1000×).

**Immunological studies.** Immunohistochemical analysis revealed that neoplastic cells were mostly and strongly positive for a B-cell phenotype CD20 (Fig. 3) and CD79a (Fig. 4), but did not stain for T phenotype CD3.

## Discussion

The WHO system defines human lymphoid neoplasms as 16 disease subtypes of B- and T-cell tumors that differ greatly in presentation, normal biology, rate of progression and response to therapy. Consequently, these neoplasms are very specifically identified and treated.^12^

Remarkably, canine lymphomas, like human, differ greatly in the natural rate of progression and response to therapy. Canine lymphomas have been described in the WHO format and there is now very strong evidence that when specifically identified, they closely mimic the human counterparts in gaining remission and projected survival patterns.^12^

Valli *et al*. stated that veterinary pathologists who are not specialist in hematopathology can achieve a high degree of accuracy in applying the WHO classification system for lymphomas.^12^ In the present study, diffuse large B cell lymphoma, DLBCL-CB was diagnosed histopathologically and cytologically in the male mixed breed dog according to the WHO classification for malignant lymphomas. Based on this classification, the diffuse arrangement of sheets of neoplastic B cells, the uniformly large nuclei (> 2 red cells in diameter) and moderate cytoplasm of the neoplastic cells are the main features of DLBCL. Nuclei are usually round or rarely cleaved or indented. Mitotic rates vary, but are detectable in all fields at 40× magnification.^7^ The DLBCL was further divided according to the number and location of their nucleoli. Large B cells with multiple nucleoli, often located at the nuclear periphery, were referred to as centro-blastic (DLBCL-CB). Those with a single central prominent nucleolus were termed immunoblastic (DLBCL-IB).^7^

Although, our case had both types of nucleolar arrangements, it was only assigned to DLBCL-CB because at least 90.0% of the nuclei were of that type.

The FNA in the initial diagnosis of human lymphomas became more widely accepted only in the 1990s, when ancillary studies (especially immunophenotyping) became routinely used in the diagnosis of lymph node aspirates suspected of lymphoma, and the classification of lymphomas was modified with more emphasis placed on cyto-morphology (rather than histologic/architectural pattern), immunophenotypic, and cytogenetic features in the revised European American lymphoma (REAL) classification of 1994, and the WHO classifications of 2001 and 2008.^13^

Although a major disadvantage of FNA is the lack of information concerning the morphological structure of the lesion and it is not possible to provide a specific diagnosis of many of the lymphoma subtypes based on cytological assessment, FNA biopsy is an inexpensive and rapid procedure, providing less inconvenience and discomfort to the patient, permitting multiple-site biopsy, and allowing serial sampling, particularly when inconclusive results are obtained.^12^^,^^14^

The DLBCL may be confused with Burkitt-like lymphoma (BKL). In human, BKL is a lymphoma that morphologically resembles Burkitt lymphoma, but has more pleomorphism or larger cells than classical Burkitt lymphoma, and has a proliferation fraction of > 99.0%. The pathologists proposed to define BKL as a subtype of large B-cell lymphoma. However, there was a clear consensus among the oncologists that this would be a mistake.^6^^,^^10^

According to Valli *et al*.,^10^ the standard criteria for BKL are if there are large cells with nuclei two times or larger than red cells in diameter in every field, then the diagnosis is large cell type despite 90.0% of the cells being of intermediate size (nuclei are 1.5 fold the diameter of RBC).^12^

Furthermore, DLBCL must be distinguished from marginal zone lymphoma, which has a characteristic arrangement of neoplastic B cells around atrophic fading follicles, intermediate-sized rather than large nuclei, more uniformly abundant cytoplasm, and an absence of mitotic figures in most cases. DLBCL must also be differentiated from large T cell lymphoma that may appear identical except for immunophenotype. Late-stage (grade III) follicular lymphoma may be similar cytologically but is distinguished on the basis of the uniformly diffuse architecture present in DLBCL.^7^

For the diagnosis of different types of lymphoma and also to differentiate between lymphoma and other similar tumors, immunohistochemistry is a useful method. Diagnostically, important CD antigens for the characterization of malignant lymphoma include CD3 and CD79. The CD3 is a complex of five polypeptides associated with the T-cell receptor (TCR). Demonstration of the CD3 antigen on malignant lymphocytes identifies the lymphoma as being of T-lymphocyte origin. Similarly, CD79 exists as a heterodimer associated with the B-cell receptor (BCR) and is required for B-lymphocyte intracellular signal transduction. Demonstration of the CD79 antigen on malignant lymphocytes delineates the malignant lymphoid population as being of B-lymphocyte origin. Rarely, malignant lymphocytes fail to demonstrate either CD3 or CD79 antigens. In such instances, the cell of origin cannot be determined, and these lymphomas are classified as being derived from null cells.^15^^,^^16^

The CD20 is a tetra-span-transmembrane phosphor- protein that is expressed predominantly in pre-B cells and in mature peripheral B cells in humans and mice. It has been shown that the antibody that recognizes the intracellular domain binds a canine CD20 homolog and is suitable for use as part of a panel to identify normal and malignant canine B cells in routine diagnostic samples.^15^^,^^16^

The WHO human classification has been adapted to veterinary medicine. However, further cooperative research programs between clinicians and pathologists needed to ascertain the classification. The considerable range in survival times is associated with the fact that lymphoma subtypes have different prognoses and require different treatments. Knowledge of the specific lymphoma subtypes is therefore necessary to select an adequate treatment, in order to reduce potentially unnecessary suffering of the patient due to the side effects of chemotherapy as well as save costs for the owner.^17^^,^^18^

In conclusion, in spite of our little experience in lymph node pathology, we could simply use the criteria of the WHO classification for the diagnosis and classification of canine lymphomas. The WHO classification system is valuable to veterinary pathology since it permits the application of oversight criteria and allows the addition of newly derived information. Furthermore, similar to human lymphomas, a histopathological diagnosis with immuno-phenotyping is a minimal requirement for diagnosis of specific subtype and selecting the most appropriate chemotherapy.
